# Assessment of oxidative stress response genes in *Avicennia marina* exposed to oil contamination – Polyphenol oxidase *(PPOA)* as a biomarker

**DOI:** 10.1016/j.btre.2020.e00565

**Published:** 2020-11-20

**Authors:** Babak Moradi, Ralph Kissen, Hassan Zare Maivan, Mehri Seyed Hashtroudi, Mona Sorahinobar, Torfinn Sparstad, Atle M. Bones

**Affiliations:** a Department of Plant Biology, Faculty of Biological Sciences, Tarbiat Modares University, Tehran, Iran; b Cell Molecular Biology and Genomics Group, Department of Biology, Norwegian University of Science and Technology (NTNU), Trondheim, Norway; c Iranian National Institute for Oceanography and Atmospheric Science, Tehran, Iran; d Department of Plant Biology, Faculty of Biological Science, Alzahra University, Tehran, Iran

**Keywords:** Mangroves, Oil pollution, Gene expression, Detoxification, Polycyclic aromatic hydrocarbons (PAH), Biomarker

## Abstract

Mangrove plants, which inhabit and form sensitive ecosystems in the intertidal zones of tropical and subtropical coastlines, though vulnerable to petroleum pollution, still maintain their growth under oil contamination. To elucidate the molecular response of mangrove plants to crude oil–sediment mixture, seeds of *Avicennia marina* were planted and grown on 0, 2.5, 5.0, 7.5 and10 % (w/w) oil-contaminated soil. Plant biomass was highly affected from 3.05 ± 0.28 (Control) to 0.50 ± .07 (10 %) and from 3.47 ± 0.12 to 1.88 ± 0.08 in 2 and 4 months old plants respectively. The expression analysis of 11genes belonging to detoxification pathways in the roots and leaves of 2 and 4 month-old plants was evaluated by qRT-PCR. Our results showed changes in expression levels of *Fe-SOD*, *Mn-SOD*, *CAT*, *PRX*, *PPO*s, *GST*s, and *NAP*2 whose products are involved in reactive oxygen species (ROS) and xenobiotic detoxification. *PPOA* showed the highest expression induction of 43 ± 1.15, followed by *CAT* (12.61 ± 3.25) and *PPOB* (6.38 ± 1.34) in leaves of 2 months old seedlings grown on 7.5, 10 and 7.5 % oil contaminated soil respectively. *PPOA* (39.23 ± 2.1), *PRX* (32.13 ± 1.2) as well as *PPOB* (26.11 ± 1.3) showed the highest expression induction in leaves of 4 months old plants grown in 2.5 % oil contaminated soil. Our data indicated that PPOA can be a good biomarker candidate gene for long term exposure to oil contamination in *A. marina*.

## Highlights

• *Avicennia marina* plants tolerate exposure to mild oil contamination in soil and they can survive at higher concentrations.
• Oil contaminated soil induced stronger transcriptional responses in leaves than in roots of *A. marina*.
• *Our suggested biomarker PPOA* showed a significant up-regulation in leaves under all tested oil concentrations

## 1 Introduction

Mangrove ecosystems are essentially vital and adaptable coastline environments, which house a wide diversity of organisms. They stabilize coastlines through their branches and roots which also trap excess deposits, filter water to a better quality favorable for the growth of coral reefs, and greatly reduce coastal erosion by dissipating the energy from incoming waves [1]. The Iranian mangrove forests, which are primarily comprised of *Avicennia marina*, are located in coastal zones of the Persian Gulf and the Gulf of Oman over a range of 1830 km from east to west in southern Iran [2].

Mangrove ecosystems throughout the world face several threats, including pollution, deforestation, fragmentation, and sea-level rise [3]. These ecosystems are highly vulnerable to oil spills and show a range of stress responses and even lethal effects following oil exposure [4]. Bioindicator species are known as species whose function, population, or status can reveal the qualitative status of the environment. *A. marina* as a prevalent plant species of the Persian Gulf mangrove ecosystems can be used as a bioindicator for environmental pollution in the coastal environment. Biological monitoring of natural ecosystems can be performed by the use of biomarkers from bioindicator species to quantify the degree of exposure to contaminants [5]. Biomarkers are very important in different fields of biological science [6,7] and different biochemical and genetic methods are used to find the best biomarkers [8,6]. A pollution responsive biomarker is a quantitative measure of changes in molecular or cellular components, processes, structures and functions related to exposure to environmental chemicals [9]. Results of the current study can help finding a biomarker gene for oil contamination.

Oil is a complex mixture of different toxic and harmful chemicals that consists of a variety of hydrocarbon-based substances such as alkanes, cyclo-hexanes, and polycyclic aromatic hydrocarbons (PAHs) [10]. Research on the physiological and biochemical responses in mangrove plants to oil contamination has been conducted [11,12], while not much attention has been paid to gene regulation. Some studies with oil components have been performed [13,14] and a hypothesis that oil and PAHs contaminations result in oxidative stress in plants has been suggested [[15], [16], [17]]. Additionally, an increase in the activity of scavenging antioxidant enzymes of mangrove plants in response to oil contamination has been reported [18,19]. To assess if these changes are the result of alterations in the expression of genes, we identified relevant genes and studied their regulation in response to oil contamination. This will allow us to better understand the underlying mechanisms and to screen for bio-indicators for environmental pollution in the future. Genes putatively encoding superoxide dismutases (*SODs*), catalase (*CAT*), peroxidase (*PRX*), ascorbate peroxidase (*APX*), polyphenol oxidases (*PPOs*), glutathione-S-transferases (*GSTs*) and non-intrinsic ABC protein 2 (*NAP2*) were selected as they are an indication of stress responses. To the best of our knowledge this is the first study in which the molecular response of *A. marina* to oil contamination was investigated. The findings of the current study, in addition to providing a basal knowledge on responses of *A. marina* genes related to antioxidant enzymes to long-term oil pollution examined the possibility of using them as biomarkers for oil contamination.

## 2 Material and methods

### 2.1 Soil properties and crude oil treatment

Soil was collected from the A horizon of Bagho Nursery site in Bandar Abbas, Hormozgan, Iran. The soil pH was 7.9 and its texture was sandy loam. Soil samples were sieved through a 2 mm mesh, and then sterilized at 121 °C for 2 h. Crude oil (Table1), obtained from Tehran Reﬁnery, was added and mixed with soil thoroughly at concentrations of 2.5, 5.0, 7.5, and 10.0 % (w/w). The control treatment consisted of the same soil that was not mixed with crude oil.

**Table 1:** Effect of 10 % (w/w) oil contamination on the content of polycyclic aromatic hydrocarbons (PAHs) in soil (ng/g).

PAH	Structure	formula	Control	10 % (w/w) oil-contaminated soil
Naphthalene		C_10_H_8_	139.97 ± 3.73	6011.2 ± 6.7
Acenaphthylene		C_12_H_8_	246.42 ± 3.47	2098.0 ± 1.7
Acenaphthene		C_12_H_10_	204.13 ± 3.94	1123.0 ± 6.8
Fluorene		C_13_H_10_	219.59 ± 2.21	2830.0 ± 24.5
Phenanthrene		C_14_H_10_	75.31 ± 2.23	4978.7 ± 20.6
Anthracene		C_14_H_10_	ND	120.0 ± 5.3
Fluoranthene		C_16_H_10_	3.34 ± 0.25	4300.0 ± 10.0
Pyrene		C_16_H_10_	34.19 ± 1.22	650.0 ± 11.0
Benzo(a)anthracene or Tetraphene		C_18_H_12_	ND	3691.0 ± 5.5
Chrysene		C_18_H_12_	ND	150.0 ± 11
Benzo(b)fluoranthene		C_20_H_12_	ND	405.0 ± 16.0
Benzo(k)fluoranthene		C_20_H_12_	ND	59.0 ± 4.0
Benzo(a)pyrene		C_20_H_12_	ND	449.9 ± 13.1
Indeno[1,2,3-cd]pyrene		C_22_H_12_	ND	83.8 ± 5.5
Dibenz(a,h)anthracene		C_22_H_14_	ND	57.8 ± 3.4
Benzo(g,h,i)perylene		C_22_H_12_	ND	153.0 ± 5

### 2.2 PAHs assessment

Non-treated control soil and 10 % (w/w) oil-spiked-soil were sampled at the start of the experiment and were used for PAH analysis. The extraction method was adapted from MOOPAM 2010 with some modifications. Briefly, three replicates of 2 g of freeze-dried soil sample were extracted with dichloromethane: acetone (1:1) under sonication, and the solvent was reduced under vacuum with a rotary evaporator. The extract was cleaned up on activated copper for sulfur removal and a silica-alumina column with hexane and hexane-dichloromethane (90:10) as eluents. After removal of the solvent, the final residue was dissolved in 1 mL hexane. Analysis of PAH was performed with an Agilent 6890 N GC system equipped with a 5973 mass detector and an MSD Chemstation software on an HP-5 fused silica capillary column (30 m ×0.25 mm ×0.25 μm).

### 2.3 Plant growth conditions

Mature and uniform propagules of *Avicennia marina* were collected from Tāsbar Creek of Bandar Abbās-Hormozgan, surface sterilized with 1 % sodium hypochlorite in water for 10 min, and washed thoroughly in sterilized distilled water. Two healthy propagules were sown equidistantly in pots. A total of 25 pots (i.e. 50 plants) were used for each of the five treatments (control and four oil concentrations). Plants were irrigated with 100 mL of water every alternate day. All experiments were carried out in a greenhouse under a temperature regime of 21 and 18 °C during the day and night, respectively. Fresh and dry weights of three biological replicates (each consisting of tissue pooled from 10 plants) were determined on 60 and 120 days after planting.

### 2.4 Quantitative real-time PCR (qPCR) gene expression analysis

At two time points, 60 and 120 days after planting, all roots and leaves tissues were harvested separately from individual plants and frozen immediately in liquid nitrogen. Samples were freeze-dried (freeze-drier model: OPR-FDB-5503, Korea) and tissue from 10 plants was pooled to generate one biological replicate. RNA was isolated using the Spectrum Plant Total RNA kit (Sigma –Aldrich) following the manufacturer’s protocol. An on-column DNase treatment was performed using the RNase-Free DNase set (Qiagen). Total RNA was quantified using a NanoDrop NP-1000 spectrophotometer (NanoDrop Technologies). RNA integrity was checked on a 2100 Bioanalyzer (Agilent). All samples had RNA integrity number (RIN) values above 8. cDNA was synthesized from 1 μg total RNA using the QuantiTect Reverse Transcription Kit (Qiagen) and diluted 10 times in ddH_2_O. Quantitative real-time PCR (qPCR) was performed using the Light Cycler 480 SYBR Green I Master (Roche) on a LightCycler96 system (Roche) programmed as follows (1) preincubation at 95 °C for 5 min, (2) 40 cycles of amplification consisting of 95 °C for 10 s, 55 °C for 10 s and 72 °Cfor 10 s, and (3) melting curve analysis by heating from 65 °C to 97 °C with a ramp rate of 2.2 °C/s. Each 20 μL reaction contained 0.5 μM of each of the forward and reverse primer (Table 2). When possible, primers were designed on annotated *Avicennia marina* sequences. Otherwise, primers were designed on *Avicennia marina* sequences that were identified by BLAST with annotated *Arabidopsis thaliana* genes on the gene databases and sequence read archive (SRA) of NCBI (https://www.ncbi.nlm.nih.gov) and the Mangrove Transcriptome Database (http://mangrove.illinois.edu/transcriptome). Quantification cycle (Cq) values for each amplification curve were determined by the LightCycler 96 software version 1.1 (Roche). LinRegPCR software [20,21] was used to determine the mean PCR efficiency for each primer pair. After analyzing the stability of the genes selected as possible reference genes by geNorm [22] in qbase+ *ACT2*, *UBQ10,* and *TIP41-like* were chosen as reference genes (while excluding *PP2AA3*) providing a more accurate normalization compared to the use of a single non-validated reference gene.

**Table 2:** Primers used for qRT-PCR of reference and target genes.

	Accession number (where available)	Gene	Product	Forward primers(5′- 3′)	Reverse primers (5′- 3′)
Target Genes	EU025130.1	*APX1*	Ascorbate peroxidase	GCAATACTGGTGACAAAGTGC	TCGTACAAGTAACTCAGGATCACC
	AY272049.1	*CAT*	Catalase	ATGGGTCGACGCTTTATCTG	TTGTCGGCCTTACATTGAGG
	AF328859.1	*Cu/Zn-SOD*	Cu/Zn-Superoxide dismutase	AGGACCACATTCCATAGTTGG	GAAGACCAATGATACCACAAGC
		*Fe-SOD*	Fe-Superoxide dismutase	CTGGGATTATTCTCCGCTGC	CATCCCAAGAAACAAGATTCTCC
	AY137205.1	*Mn-SOD*	Mn- Superoxide dismutase	GCCTTTGCTTGGTATTGATGTC	CATAAACTTCACTGGCGTATTTCC
		*PPOA*	Polyphenol Oxidase	AAGTCCACAACTCCTGGCTG	CCCAGGATTCTTTCGAAGAAG
		*PPOB*	Polyphenol Oxidase	GGCTTTTCTTTCCCTTCCAC	GCGAAAGTGGGGTCATTTATC
	AB049589.1	*PRX*	Peroxidase	CAACTAGCCACGGACAAGAGG	TCTCGGACAGAACGGTGATG
		*GSTU4*	Glutathione-S-Transferase	GAAGGTGCCTGTTCTTGTGC	GCTTTCTCGTACGGATCTTGG
		*GSTU25*	Glutathione-S-Transferase	TGGAGACAAGACTTACTTTGGAGG	TGCTGAAGTTGCCAAAAGTCTC
		*NAP2*	Non-intrinsic ABC protein	TTGATGGACTGGAGTCTTGG	GCCAAGATTCAACAACAGATAGC
Reference Genes		*ACT2*	Actin 2	GTGTGATGTGGATATCAGGAAGG	CCTTAATCTTCATGCTGCTT
		*PP2AA3*	Protein phosphatase	GCAAATTCTACCCTGTGTAAAGG	CTCAATTGTTGCATCCTTCC
		*TIP41-like*		AGATGAGTTGGCTGACAATGG	ACTCCATCAACTCTGAGCCAG
		*UBQ10*	polyubiquitin 10	GCAAGACCATCACTCTCGA	GCTTTCCAGCGAAGATCAGC

### 2.5 Statistical analyses

Statistical analysis of the effect of oil on morphological variables and gene expression ratios in leaves and roots of oil treated samples compared to control samples was performed with Graphpad Prism v.8 (GraphPad, USA) and qbase + version2.6.1 [23] respectively. Heatmap correlation analysis was performed using MetaboAnalyst web portal (https://www.metaboanalyst.ca/). Principal component analysis (PCA) was conducted using publicly available Past3.16 software.

## 3 Results

### 3.1 Effect of oil contamination on fresh and dry weight of *Avicennia marina*

The contamination of soil with crude oil caused an increase in the total PAHs in soil samples at the beginning of the experiment (Table 1). In soil polluted with 10 % oil, the sum of PAHs increased about 30-fold relative to the control (not contaminated). Among the PAHs, two-ringed naphthalene showed the highest increase with oil contamination.

The fresh and dry weight of *A. marina* seedlings decreased significantly under oil contamination (Table 3). Plant seedlings showed significantly greater root biomass in the 2.5 and 5 % w/w soil contaminated treatments as compared with the control over the growth period. Leaf biomass decreased significantly in all treatments as compared with the control. In the presence of oil, the shoot/root ratio changed in favor of greater root production. Some plants exhibited a lack of shoot initiation and growth at 10 % oil contamination as they developed roots but not shoots.

**Table 3:** Changes in fresh and dry weight (in g) of *A. marina* after exposure to different concentrations of crude oil (n≥10 seedlings).

			Control	2.5 %	5.0 %	7.5 %	10.0 %
2 months old plants(g)	**Fresh Weight**	**Root**	1.56 ± 0.23a	1.97 ± 0.36a	1.68 ± 0.44ab	1.14 ± 0.45c	0.41 ± 0.1d
		**Shoot**	1.49 ± 0.33a	0.88 ± 0.11b	0.69 ± 0.34bc	0.55 ± 0.13c	0.09 ± .05d
		**Leaf**	0.95 ± 0.24a	0.42 ± 0.13b	0.48 ± 0.11b	0.15 ± 0.04c	0.12 ± 0.03c
		**Shoot/Root**	1.54 ± 0.28a	0.69 ± 0.2b	0.67 ± 0.29b	0.64 ± 0.12b	0.47 ± 0.12b
	**Dry Weight**	**Root**	0.27 ± 0.04bc	0.35 ± 0.06a	0.32 ± 0.06ab	0.23 ± 0.09c	0.10 ± 0.07d
		**Shoot**	0.34 ± 0.07a	0.19 ± 0.02b	0.13 ± 0.03c	0.10 ± 0.03c	0.02 ± 0.01d
		**Leaf**	0.2 ± 0.01a	0.1 ± 0.01bc	0.12 ± 0.02b	0.04 ± 0.04c	0.04 ± 0.04c
		**Shoot/Root**	2.00 ± 0.15a	0.82 ± 0.24b	0.76 ± 0.1b	0.62 ± 0.2b	0.59 ± 0.1b
4 months old plants(g)	**Fresh Weight**	**Root**	1.76 ± 0.07bc	2.22 ± 0.11a	1.99 ± 0.19ab	1.41 ± 0.12 cd	1.21 ± 0.10d
		**Shoot**	1.71 ± 0.17a	1.06 ± 0.07b	0.8 ± 0.07bc	0.62 ± 0.07c	0.59 ± 0.06c
		**Leaf**	1.46 ± 0.12a	0.61 ± 0.10b	0.57 ± 0.10bc	0.34 ± 0.05 cd	0.28 ± 0.05d
		**Shoot/Root**	1.89 ± 0.15a	0.77 ± 0.14b	0.70 ± 0.1b	0.69 ± 0.12b	0.74 ± 0.11b
	**Dry Weight**	**Root**	0.29 ± 0.04b	0.38 ± 0.08a	0.37 ± 0.1a	0.28 ± 0.07b	0.24 ± 0.06b
		**Shoot**	0.38 ± 0.11a	0.2 ± 0.09b	0.15 ± .04bc	0.11 ± .0.04c	0.1 ± 0.03c
		**Leaf**	0.3 ± 0.08a	0.13 ± .0.08b	0.13 ± 0.07b	0.08 ± .0.04c	0.07 ± 0.04c
		**Shoot/Root**	2.36 ± 0.1a	0.87 ± 0.27b	0.78 ± 0.24b	0.67 ± 0.28b	0.71 ± 0.2b

Values in each line marked with the same letter do not differ significantly at p ≤ 0.05.

### 3.2 Gene expression changes in *Avicennia marina* in response to oil exposure

Plants induced specific gene responses, depending on the treatment they were exposed to. The expression patterns of 11 genes: *Mn-SOD*, *Fe-SOD*, *Cu/Zn-SOD*, *CAT*, *PPOA*, *PPOB*, *APX1*, *PRX*, *GSTU4*, *GSTU25* and *NAP2*, which belong to antioxidative and detoxification pathways were assayed by qPCR in leaf and root tissues of 2 and 4 months old *A. marina* grown on soil contaminated with different concentrations of oil. geNorm analysis on the stability of putative reference gene expression revealed that *ACT2*, *UBQ10* and *TIP41-like* were suitable reference genes for the assessment of antioxidative enzyme gene expression of *A. marina* in response to oil contamination.

### 3.3 Gene expression changes in leaves of plants exposed to oil

**Fig. 1:**
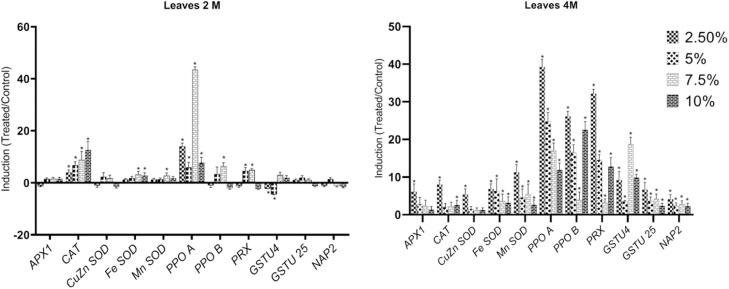
Changes in transcriptional level of selected genes in leaves of two and four months old *A. marina* exposed to different levels of oil contamination. Means SEs, n = 3, *-significant differences between the control and treated samples (*P ≤ 0.05*; *t-test*).

In leaves of 2 months old plants two of the 11 selected genes, *PPOA* and *CAT* were induced significantly (*p < 0.05*) by all treatments compared with control plants (Fig. 1). The expression of five other genes: *PPOB*, *Mn-SOD*, *Fe-SOD*, *PRX* and *GSTU4* showed a significant induction by at least one of the treatments, with 7.5 % oil affecting most of them. No significant differences in gene expression were observed in leaves for *Cu/Zn-SOD*, *GSTU25* and *NAP2* at any of the assayed concentrations of crude oil. *GSTU4* was the only gene whose transcript level was significantly reduced by oil exposure in two months old leaves (Fig. 1).

After 4 months of oil exposure, 8 of the 11 selected genes were significantly (*p < 0.05*) induced in leaves by all treatments compared with control plants (Fig. 1), with *PPOA*, *PPOB* and *PRX* showing particularly high induction levels. In addition, the expression of *CAT*, *Cu/Zn-SOD,* and *APX1* showed a significant induction by the 2.5 % oil treatment.

### 3.4 Gene expression changes in roots of plants exposed to oil

**Fig. 2:**
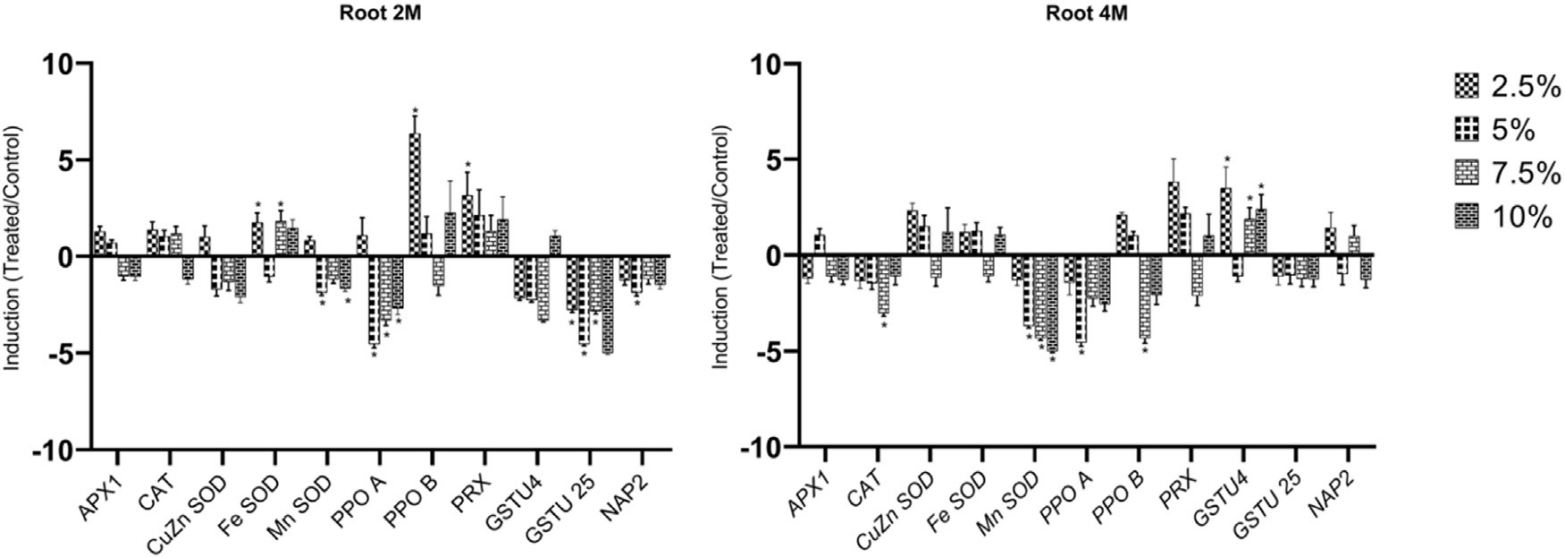
Changes in transcriptional level of selected genes in roots of two and four months old *A. marina* exposed to different levels of oil contamination. Means SEs, n = 3, *-significant differences between the control and treated samples (*P ≤ 0.05*; *t-test*).

In the roots of two months old plants, the expression of *GSTU25* was significantly reduced in all treatments (Fig. 2). Four other genes (*GSTU4*, *PPOA*, *Mn-SOD*, *NAP2*) were repressed by at least one of the oil treatments, most by the 5 % oil exposure. No significant differences were observed in gene expression of *CAT*, *APX1* and *Cu/Zn-SOD* under any of the treatments. Only a few genes were significantly induced in two months old roots, such as *PPOB*, *PRX* and *Fe-SOD,* which were induced 6.37, 3.16 and 1.75 fold, respectively by the 2.5 % oil treatment

Among the 11 genes that were assessed, the six genes *PRX*, *GSTU25*, *APX1*, *Fe-SOD*, *NAP2,* and *Cu/Zn-SOD* were not significantly affected by any of the oil treatments in roots of 4 months old plants (Fig. 2). The expression of four genes was reduced under at least one treatment: *Mn-SOD* under 5 %, 7.5 % and 10 %, *PPOB* and *CAT* under 7.5 % and PPOA under 5 % oil contamination. Only the expression of *GSTU4* was significantly induced in the roots of four months old plants exposed to oil (Fig. 2).

**Fig. 3:**
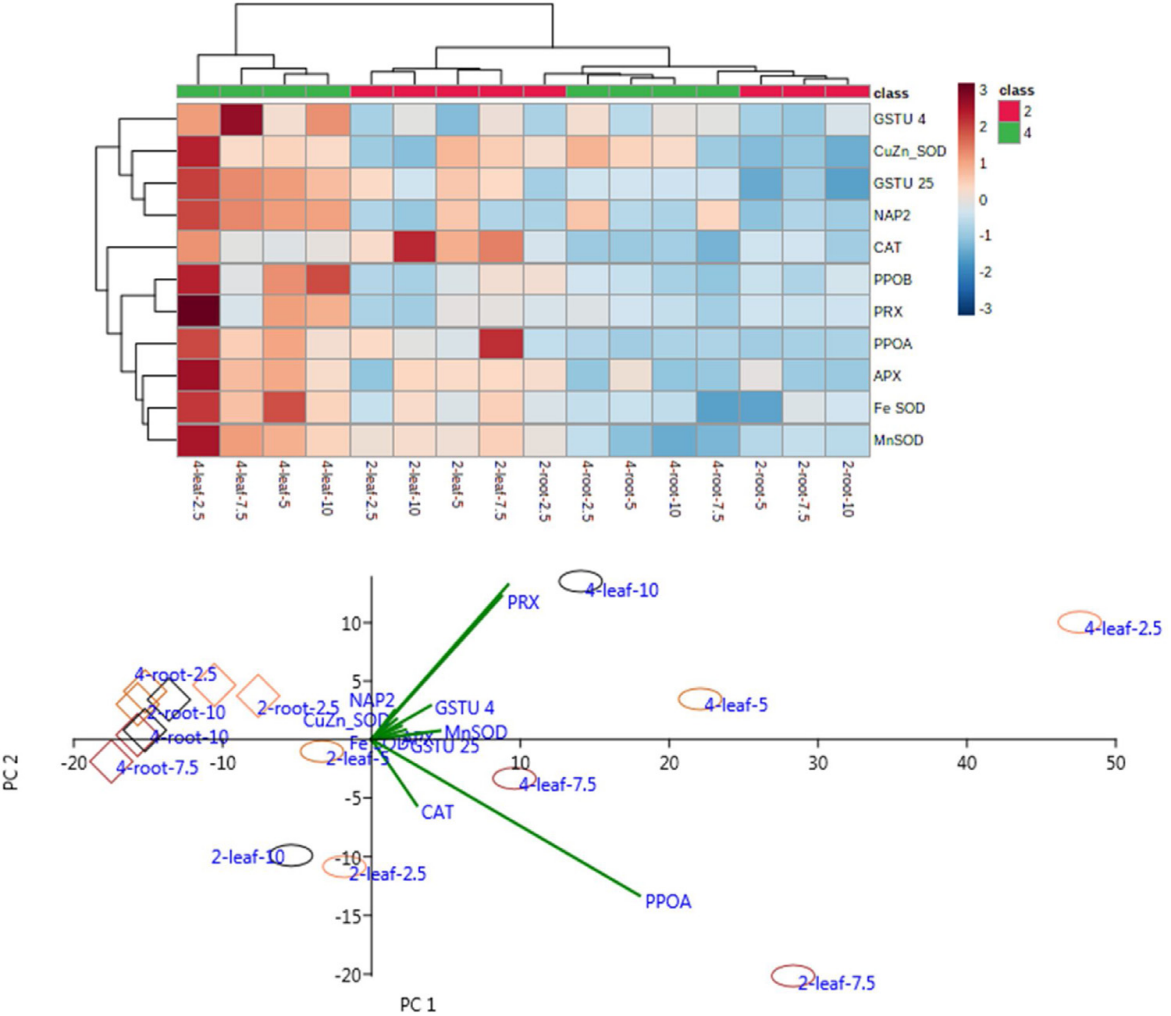
Heatmap and PCA analysis of the change in transcript levels of the selected genes in two and four months old root and leaf of *A. marina* grown on 2.5 to 10 % (W/W) oil contaminated soil.

Heatmap and PCA analysis of the induction level of the selected genes of *A. marina* seedlings grown on oil contaminated soils showed that under the four levels of oil concentration, leaves and roots showed a completely different response (Fig. 3). Changes in transcript levels of *PRX* and *PPOB* (Pearson correlation coefficient or PCC = 0.92), Mn-SOD and *Fe-SOD* (PCC = 0.87), and *APX* and *Mn-SOD* (PCC = 0.86) showed a strong correlation under oil contamination.

The PCA of transcriptional changes of the selected genes showed that about 90 % of variation between treatments could be explained by two principal components (Fig. 3b). The first component (PCA1) separated the four months old leaves from other samples primarily based on PPOA, PPOB and PRX expressional changes (Fig. 3b) (x-axis). Treated two and four months old root samples completely separated from the leaves by PC1. The second component (PCA2) separated mostly two months old leaves samples from the other samples, which was mainly based on PPOA, PPOB and PRX expression levels (y-axis).

## 4 Discussion

Petroleum contamination of the rhizosphere affects plant functions both physically by attaching to roots and through dissipation of volatile compounds. Coating blockage of crude oil on roots surface may cause low water accessibility and oxygen deficiency [24]. Under water deficiency, plant growth is readily inhibited and growth of roots is favored over that of leaves [25]. Previous reports showed that the presence of petroleum hydrocarbons can be toxic and significantly reduce plant biomass [[26], [27], [28]]. In our study, growth inhibition of crude oil on *A. marina* was obvious and similar to those reported by other investigators [14,29].

Our study confirmed that *A. marina* plants tolerate mid-term exposure to mild oil contamination in soil, even though a significant decrease in plant growth was observed at higher concentrations. The enhanced root growth at lower oil concentrations may be due to a stress response [30] or be a strategy for the plant to stimulate water and nutrient uptake [31].

Oil contamination is known to be one of the main abiotic stress types for mangroves and leads to the production of reactive oxygen species (ROS) [18,24]. Reports have documented that oil contamination provokes an increase in cellular levels of ROS, leading to oxidative damage enhancing the stress in plants [18,32,33]. Nonetheless, plant cells have developed different strategies such as enzymatic and non-enzymatic defense systems in order to mitigate oxidative stress [34].

The excess ROS in plants seriously disrupt normal metabolism through oxidative damage to lipids, proteins and nucleic acids, and may eventually cause plant growth inhibition or even death. To respond to the oxidative stress, activities of a series of antioxidative enzymes including different forms of CAT, SOD, and PRXs in plants increase to better scavenge ROS. These changes in enzyme activities can be due to an increase in the expression level of their corresponding genes [35].

We observed overall greater changes in gene expression in leaves compared to roots, which may indicate a higher sensitivity of leaves to the oil contamination stress. Genes were also much more responsive to oil contamination in leaves of four months old plants in comparison with two months old plants, which may be because of the longer exposure to contamination. The nutrients stored in cotyledons might also have a mitigating effect on the stress [36] and this effect would be expected to be stronger in younger plants.

SOD is involved in the first step of ROS elimination by catalyzing the conversion of O_2_^−^ to H_2_O_2_ and O_2_, H_2_O_2_ being further decomposed by CAT, PRXs and APX [37]. Our findings of increased expression levels of *SODs, PRX* and *APX*, especially in the leaves of four months old plants, under oil treatment are in agreement with observations of Liu et al. [16] who reported an increase in the SOD, PRX and APX enzyme activities in *A. thaliana* under phenanthrene treatment. The observed upregulation of these genes may help plants to reduce the deleterious effects of ROS cytotoxicity. This finding is also consistent with transcriptional studies that reported an increase in *APX1* transcripts following phenanthrene treatment [16]. In our data *CAT* expression was upregulated in leaves, while its expression was not affected or even down-regulated in roots exposed to oil.

Other enzymes such as PPOs are known to catalyze the oxidative transformation of a large number of phenolic and non-phenolic aromatic compounds to their corresponding quinones which are insoluble and less toxic [38,39]. Liu et al. [40] showed for example that the rhizosphere soil PPO activity of *Echinacea purpurea* and *Festuca arundinacea* Schred increased after cultivation on PAH-contaminated soils. The induction of *PPOA* and *PPOB* in leaves of *A*. *marina* in our study may be related to their physiological function in PAH degradation process [40] and [41].

Proteomic analysis of *A. thaliana* exposed to phenanthrene indicated that antioxidant activity is the most significant term in the molecular function ontology [42]. That study also showed that phenanthrene exposure induced reactive oxygen formation and significantly altered the activities of enzymes such as CAT, APX and peroxiredoxins in *A. thaliana*. Our findings are therefore in agreement with previous physiological, transcriptional, biochemical and proteomics studies in *A. thaliana* which implied oxidative stress as a major component of plant response to PAH contamination [15,16,43,44].

Youssef [14] showed a linear relationship between the PAHs doses applied to *A. marina* seedlings and the amounts accumulated in their leaf tissue. Additionally, Jia et al. [45] reported increased concentrations of phenanthrene and pyrene in *A. marina* leaves with enhancing their sediment concentrations. These observations are consistent with a significant decrease of PAHs concentration in rhizospheric soil of *A. marina* in comparison with a non-rhizospheric control (Moradi et al., under publication), an indication for plant uptake of PAHs in our assays. A transfer of PAHs from root to shoot may be the main cause of gene induction in leaves. Glutathione S-transferases (GSTs) are enzymes that conjugate the reduced form of glutathione (GSH) to xenobiotic substrates to facilitate their detoxification. They can also function as antioxidants by tagging oxidative degradation products or by acting as a glutathione peroxidase [[46], [47], [48]]. In *A. thaliana GSTU25* was induced upon exposure to oil [17]. *GSTU4* and *GSTU25* were induced by phenanthrene [44] and in roots treated with the organic fraction of oil sands process affected water [49]. Inductions of *GSTU4* and *GSTU25* in leaves of four months old *A. marina* grown on oil contaminated soil may be related to these broad roles of *GSTs.*

Coordinated changes in expression levels of *NAP2* and *GSTU25* in response to oil contamination may be related to their sequential role in the PAHs detoxification process. As the first step of detoxification, members of the cytochrome P450 family catalyze the oxidation of potentially toxic compounds, which are subsequently conjugated to a hydrophilic molecule, such as glucose, glutathione or glucuronide [50,51]. This conjugation step makes the potentially toxic compounds more hydrophilic and prevents the newly formed compounds from crossing membranes by diffusion. As mentioned above, glutathione conjugation of xenobiotics is catalyzed by various GSTs [52]. As the final step, compound-conjugates can be transported into the vacuole or apoplast by ABC transporters. This process further reduces the toxicity of the compounds [53]. In *A. thaliana*, *NAP2* is known as a gene that encodes a member of the *NAP* subfamily of ABC transporters. Upregulation of *NAP2* was reported in *A. thaliana* under exposure to oil and phenanthrene [15,44]. Taken together, our results of the upregulation of *GSTs* and *NAP2* genes in leaves of 4 months old *A. marina* suggest their potential roles in PAHs detoxification.

Oxidative stress-related enzymes, because of their high sensitivity, have been suggested as biomarkers for recognition of the harm induced by contaminants or other environmental stresses in plants [[54], [55], [56]]. The description of the cause–effect relationship is necessary for biomarker validation [55], although such data are still very scarce. Among the eleven genes assayed in the current study, *PPOA* showed a significant and strong (more than fivefold) up-regulation in leaves of 2 and 4 months old seedlings under all tested oil concentrations (Fig. 1). It is therefore a very good candidate for further studies as a biomarker of oil contamination in *A. marina*.

## 5 Conclusion

Our study provides the basis for the investigation of antioxidative stress responsive genes of *A. marina* to oil contamination. Due to the limited number of genes assayed in the current study further efforts are needed in order to identify robust biomarker genes. Strong induction of the genes in leaves as compared to roots in both 2 and 4 months old plants confirmed that the leaves are a better source to find biomarkers for oil contamination. Our data suggest that *PPOA* could be used as a biomarker for oil contamination in the mangrove ecosystem as its strong induction may be related to its physiological function in the PAH degradation process. Research into the possible use of *PPOA* as biomarker of oil contamination in the mangrove ecosystem of the Persian Gulf and its coastal areas, the world's largest source of petroleum and related industries, is already underway with a particular focus on Nayband Bay in Asaluyeh- the south of Iran.

## Author statement

Atle M. Bones and Hassan Zare Maivan supervised the research with the assistance of Mehri Seyed Hashtroudi; Babak Moradi and Mona Sorahinobar carried out experiments with the assistance of Torfinn Sparstad; Babak Moradi, Ralph Kissen and Mona Sorahinobar analyzed experimental results and data and wrote the draft of manuscript. All authors read and approve the manuscript.

## Declaration of Competing Interest

The authors declare that they have no conflict of interest.
